# Altered Expression of Cellular Bcl-2 in the Progression of Hamster Cholangiocarcinogenesis

**DOI:** 10.1100/2012/385840

**Published:** 2012-04-30

**Authors:** Byung-suk Jeon, Byung-IL Yoon

**Affiliations:** Laboratory of Histology and Molecular Pathogenesis, College of Veterinary Medicine and Institute of Veterinary Science, Kangwon National University, Chuncheon, 200-701, Republic of Korea

## Abstract

Bcl-2 is an intracytoplasmic and membrane-associated apoptosis suppressor, and its overexpression is closely associated with survival of malignant tumors, in particular their aggressive behavior and poor prognosis. The role of Bcl-2 is, however, still controversial in cholangiocarcinogenesis because of the discrepancies in the expression of the protein. In the present study, alteration in the expression of Bcl-2 in cholangiocarcinogenesis was investigated by studying the immunoreactivities of this protein in normal, hyperplastic bile ducts with or without dysplastic changes, and neoplastic bile duct cells from a hamster cholangiocarcinoma (ChC) model. Cytoplasmic staining, which reflects high-Bcl-2 immunoreactivity, was negative to very weak in normal and hyperplastic bile ducts without dysplastic changes, while hyperplastic bile ducts with dysplasia indicated heterogeneously strong expression. On the other hand, most of the neoplastic cells of invasive cholangiocarcinomas were negative to weak as much as the level of normal bile ducts. The results suggest that the antiapoptotic factor Bcl-2 plays a limited role in the survival of highly proliferative, potentially dysplastic bile duct cells. However, the role of Bcl-2 in biliary cancer cells was not significant.

## 1. Introduction

Apoptosis, the so-called programmed cell death or cell suicide, is a key regulatory mechanism in the differentiation and maturation of an organism and during the development of cancer. Apoptosis is also essential for removing irreparable, damaged, and transformed cells in adults [1, 2]. Apoptosis is regulated by a variety of genes, including Bcl-2 and p53. p53 induces apoptosis to protect the body against cells that behave in a discoordinated fashion or have damaged DNA. The Bcl-2 oncogene inhibits apoptosis as a generalized cell death suppressor, thus allowing the accumulation and propagation of cells containing genetic alterations [3]. Altered expression of cell-survival genes such as Bcl-2 induces a malfunction in the apoptosis-regulating machinery and is therefore closely associated with carcinogenesis [4, 5]. Earlier studies reported aberrant Bcl-2 expression in a variety of tumors, such as adenocarcinoma of the prostate, bladder carcinoma, squamous cell carcinoma of the lung, nasopharyngeal carcinoma, and breast carcinoma [6–11].

 Cholangiocarcinoma (ChC) is a highly malignant, generally fatal adenocarcinoma arising from bile-duct epithelial cells of the intrahepatic or extrahepatic biliary system. Although it is a relatively rare tumor, the incidence of ChC is increasing globally [12]. Despite advances in ChC diagnosis, surgery offers the only possibility of relatively long-term survival [13]. Nevertheless, the 5-year survival rate after curative resection is less than 30% [14]. The pathogenesis of biliary cancer remains unknown, but oxidative damage, oncogene activation, and impaired apoptosis may be involved [15]. In association with antiapoptosis, Bcl-2 overexpression has been implicated in carcinogenesis. However, data regarding Bcl-2 expression in human ChC are controversial, and the antiapoptotic mechanism in ChC cells remains unknown [8, 16–19]. Some studies have indicated that ChC expresses Bcl-2. Studies involving several hematological and solid malignancies have identified a correlation between intense Bcl-2 or Bcl-X_L_ expression and poor patient response to cancer therapy and overall prognosis. A study by Charlotte et al. [8] reported that eight of 11 cases expressed Bcl-2 and suggested that the Bcl-2 protein could be a unique ChC marker. A study by Terada and Nakanuma [20] found Bcl-2 expression in one of 20 ChCs and suggested that a role of Bcl-2 is limited in ChC. These results correspond to the findings in experimental models.

We investigated altered Bcl-2 protein expression patterns during hamster cholangiocarcinogenesis to evaluate the role of Bcl-2 using immunohistochemical analyses.

## 2. Materials and Methods

### 2.1. Tissue Samples

**Figure 1:**
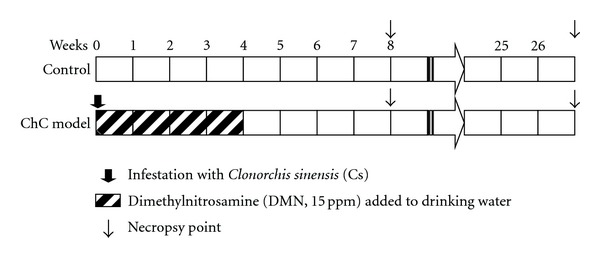
The time points of liver specimen collections in the hamster cholangiocarcinoma model. The liver specimens were respectively prepared at the precancerous phase (8 weeks after ChC model initiation) and the cancerous phase (27 weeks after the model initiation) as well as from the negative control hamsters. The hamster ChC model was composed of the initiation process by the treatment of dimethylnitrosamine (DMN) and infestation of 15 *Clonorchis sinensis *metacercariae as a strong promoter.

 Five paraffin-embedded tissue blocks of hamster livers with precancerous biliary lesions and ChC, as well as normal hamster livers, were prepared from hamster ChC models. The precancerous lesions were obtained from hamsters 8 weeks after inducing the hamster ChC model (interim phase of cholangiocarcinogenesis), and ChC tissues were obtained with tumor masses at 27 weeks (Figure 1). As described in a previous study, precancerous hamster livers were characterized by hyperplastic bile ducts with or without cellular dysplasia and intense surrounding inflammation; the ChCs were invasive tubular or tubulopapillary-type primary biliary cancers [21].

### 2.2. Bcl-2 Immunohistochemistry

 The avidin-biotin complex method was used for Bcl-2 immunohistochemistry. After dewaxing with xylene and rehydration through a graded ethanol series, tissue sections were immersed in methanol (Daejung Chemicals and Metals, Siheung City, republic of Korea) containing 0.3% hydrogen peroxide (Showa Chemicals, Tokyo, Japan) for 30 min at room temperature to block endogenous peroxidase activity. Microwave antigen retrieval was performed using a preheated target-retrieval solution (0.01 M, pH 6.0; DakoCytomation, Carpinteria, CA, USA) followed by a 15 min incubation. This was followed by treatment with a detergent, which consisted of soaking the samples in phosphate-buffered saline (PBS) containing 0.05% Tween 20 (pH 7.2). After three 5 min washes in PBS, the tissue sections were incubated with normal blocking serum and prepared according to the manufacturer's instructions supplied in the Vectastain Elite ABC Kit (Vector Labs, Burlingame, CA, USA) for 1 h at room temperature. Avidin D and biotin blocking reagents (Vector Labs) were added to the sections according to the manufacturer's instructions to minimize background staining due to endogenous biotin or biotin-binding proteins, lectins, or nonspecific binding substances present within the tissue sections. The sections were then incubated with diluted primary antibody (rabbit polyclonal anti-human Bcl-2 IgG 1 : 50, Santa Cruz Biotechnology, Santa Cruz, CA, USA) overnight at 4°C. As a negative control, PBS was used instead of the primary antibody. Thereafter, the sections were washed with PBS and incubated with the appropriate biotinylated secondary antibody according to the Vectastain Elite ABC Kit for 40 min at room temperature, followed by a 30 min incubation with ABC reagent at room temperature. The sections were visualized with 3,3-diaminobenzidine tetrahydrochloride solution (DakoCytomation) containing 3% hydrogen peroxide and counterstained with Mayer's hematoxylin (Sigma-Aldrich, St. Louis, MO, USA). This was followed by routine dehydration in alcohol, clearing in xylene, and mounting.

### 2.3. Semiquantitative Evaluation of Bcl-2 Immunoreactivity

 We counted the hyperplastic and dysplastic bile ducts in six hamster livers at 8 weeks and in 10 hamster livers at 27 weeks to semiquantitatively evaluate Bcl-2 immunoreactivity in the biliary cell populations during cholangiocarcinogenesis. Sections were evaluated at high-power magnification from a series of 10 randomly selected adjacent fields under a light microscope fitted with an ocular grid. In total, 100, 811, and 4890 biliary epithelial cells were counted in the hamster livers at normal, precancerous, and carcinogenic phases, respectively. Bcl-2 immunoreactivity in the biliary cell populations, including biliary cancer cells, was represented as the percentage of negative, weak (1+), moderate (2+), or strongly (3+) positive bile ducts or neoplastic cells for Bcl-2 to the total number of bile ducts or neoplastic cell numbers. Based on the criteria, a light microscopic examination of Bcl-2 immunoreactivity was performed blindly by two veterinary pathologists. Statistically significant differences between the biliary cell populations were detected using Student's *t*-test. Statistical significance was determined at *P* < 0.05.

### 2.4. Western Blot Assay

 As the Bcl-2 antibody used for immunohistochemistry originated from human Bcl-2, the reactivity and specificity of the primary antibody against hamster Bcl-2 were tested by western blot assay. Additionally, Bcl-2 protein levels were respectively evaluated in precancerous hamster livers, the biliary tumor mass, and in normal livers.

 Protein was extracted from the all liver tissues according to a previous study [21]. Briefly, the frozen hamster liver tissues were finely ground and dissolved by repeatedly passing them through a 26-gauge needle attached to a syringe containing protein extraction buffer (Intron, Chinju, Republic of Korea). After a 15 min incubation at 20°C, the suspension was centrifuged at 13,000 rpm for 5 min at 4°C. The protein extracts (20 *μ*g) were subjected to 12% (w/v) SDS-polyacrylamide gel electrophoresis and transferred to PVDF membranes (Bio-Rad Lab., Hercules, CA, USA). After blocking the nonspecific binding sites by incubating the membranes with 5% nonfat dried milk and 0.1% Tween 20 in Tris-buffered saline (pH 7.4) for 1 h at room temperature, the membranes were incubated overnight at 4°C with diluted primary antihuman Bcl-2 antibody at a 1 : 200 dilution, followed by an incubation with secondary antibody for 50 min at room temperature. To visualize the bands, the membranes were treated with a detection reagent for 1 min, exposed to film, and then developed using a SEER medical film processor (SEER Technologies Inc., Seoul, Republic of Korea).

## 3. Results

**Figure 2:**
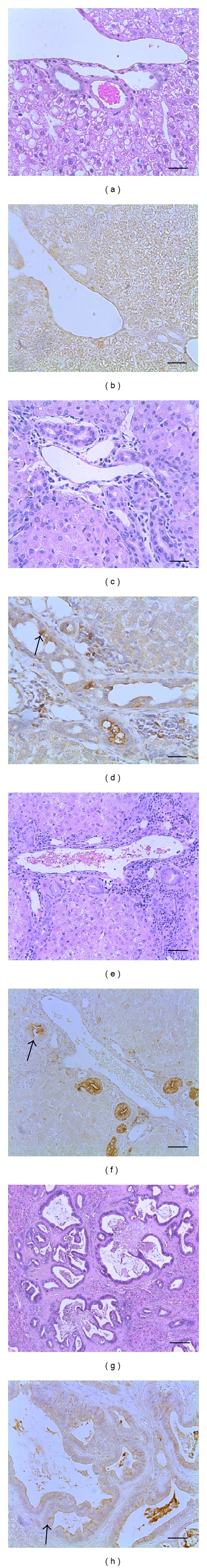
Immunohistochemistry for Bcl-2 in the liver tissue sections taken from cholangiocarcinoma (ChC) model and control group animals. (a) and (b) Normal bile ducts; (c) and (d) hyperplastic bile ducts; (e) and (f) dysplastic bile ducts; (g) and (h) cholangiocarcinoma. Bcl-2 staining was negative or weakly positive in normal and hyperplastic bile duct cells. Characteristically, Bcl-2 was heterogeneously expressed in the tissues (arrow in (d)). The Bcl-2 protein was strongly expressed in the cytoplasm of dysplastic biliary cells, although Bcl-2 expression was heterogeneous (arrow in (f)). Bcl-2 expression was negative or very weak in ChCs, similar to control levels (arrow in (h)). (a), (c), (e), and (g) hematoxylin and eosin stain; (b), (d), (f), and (h) Bcl-2 immunohistochemistry; (a)–(d) bars = 22.5 *μ*m; (e)–(h) bars = 45 *μ*m.

**Figure 3:**
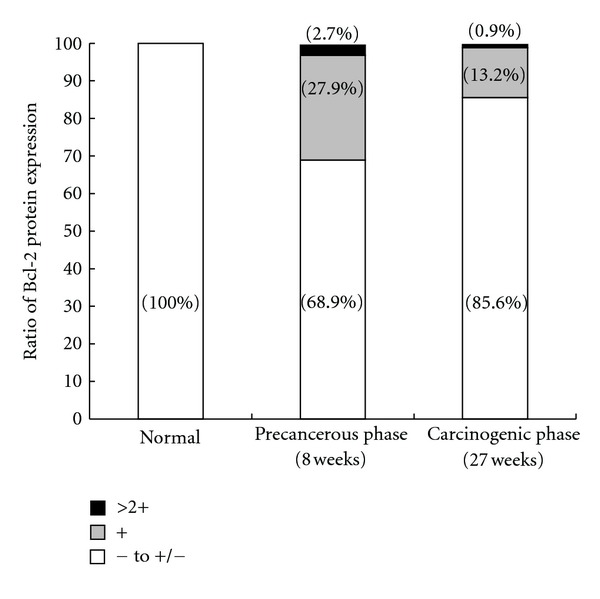
Semiquantitative analysis of Bcl-2 immunoreactivity in biliary cell populations at the precancerous and carcinogenic phases of cholangiocarcinogenesis in hamsters. The percentage of normal biliary cell populations with different grades of Bcl-2 immunoreactivity during cholangiocarcinogenesis in hamsters. The numbers on the columns represent the percentages of Bcl-2-positive cells. In total, 100, 811, and 4890 bile ducts were counted in the hamster livers at the normal, precancerous, and carcinogenic phases, respectively. Strong Bcl-2 immunoreactivity was found in some hyperplastic bile ducts with dysplastic changes at the precancerous phase. However, only <1.0% of neoplastic cells of cholangiocarcinomas indicated notable expression of Bcl-2. Normal and hyperplastic bile ducts without dysplastic changes were negative to very weak positive for Bcl-2.

High Bcl-2 expression was limited in some hyperplastic bile ducts with dysplastic changes during hamster cholangiocarcinogenesis. Most of the hyperplastic bile ducts were almost negative to weakly positive (Figure 2), and only 2.7% of the counted hyperplastic bile ducts showed high expression graded as >2+ (Figure 3). The hyperplastic bile ducts strongly expressing Bcl-2 were those with dysplastic changes, whereas the hyperplastic bile ducts without morphological transformation were all negative or very weakly positive (Figure 2). Hepatocytes in the precancerous hamster livers were generally negative. Sinusoidal, lymphocytes, eosinophils, macrophages, and plasma cells often showed strong Bcl-2 immunoreactivity.

The percentage of biliary cancer cells showing high Bcl-2 expression (above grade 2+) was much lower; it was less than 1% of the counted cancer cells in all of the ChCs examined (Figure 3). Hepatocytes in the cancerous phase did not express Bcl-2. In the normal hamster liver, all liver parenchymal cells including biliary epithelial cells and hepatocytes were negative for Bcl-2 (Figure 2).

**Figure 4:**
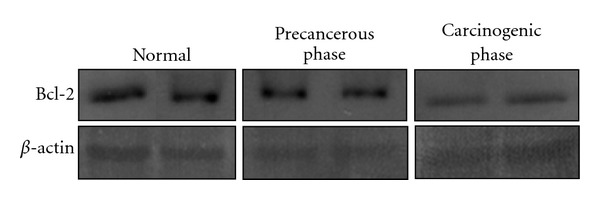
Western blot analysis for Bcl-2 in normal, precancerous, and neoplastic liver tissues in a hamster cholangiocarcinoma model. Note the specific bands made by the same antibody used for immunohistochemistry. No notable changes in the expression of Bcl-2 protein were found in the precancerous and cancerous liver tissues. The Bcl-2 expression was, compared to that of normal liver, more likely reduced in ChC tissues, which was in accordance with the results of immunohistochemistry.

 We validated the specificity of the Bcl-2 antibody used for immunohistochemistry using a western blot assay because the antibody originated from human Bcl-2. As shown in Figure 4, clear bands were noted at 21 kD, corresponding to that of the Bcl-2 protein. The protein was prepared from the whole liver tissue which includes not only bile duct cells but also hepatocytes and other liver cell components. Thus, the results of western blotting could not absolutely reflect the expression of Bcl-2 in bile duct cells, but no notable changes in expression were found in precancerous liver tissues or in the ChC samples. Rather the expression of Bcl-2 was likely reduced in the ChC tissues, compared to normal tissues, which was a similar expression pattern to that of immunohistochemistry.

## 4. Discussion

 Bcl-2 is a well-known general apoptosis suppressor that induces the formation and development of carcinomas and suppresses apoptosis. Bcl-2 is a critical factor in carcinogenesis [1], and Bcl-2 overexpression has been implicated in various tumors [6, 7, 9, 10, 19, 22]. However, Bcl-2 expression is controversial in normal liver cell components as well as in biliary cancers [8, 19]. A study by Charlotte et al. [8] reported the expression of the protein in epithelial cells of the intrahepatic small ducts, but not in hepatocytes or large bile duct cells, whereas a study by Okaro et al. [19] found no positive immunoreactivity in any of the biliary cell populations. Similarly, conflicting results have also been reported for Bcl-2 expression in association with ChC. Bcl-2 immunoreactivity occurred in more than 80% of the human ChC cases examined by Charlotte et al. [8], whereas no positive signal was found by Okaro et al. [19].

 In the present study, the immunohistochemical Bcl-2 expression patterns were compared in samples obtained from hamster livers at serial cellular stages of cholangiocarcinogenesis as well as from normal livers. The results indicated heterogeneously strong expression of Bcl-2 in dysplastic bile ducts but negative to weak immunoreactivity in normal and hyperplastic small bile ducts. Despite such heterogeneous Bcl-2 expression in the biliary cell populations, overexpression of the protein in a limited number of dysplastic bile ducts suggests that it plays a role in ChC through survival of the transformed bile duct cells, which are presumably candidates for developing ChC. A high concentration of the antiapoptotic protein Bcl-2 within the biliary tree might allow cells that harbor growth promoting mutations, such as K-ras, c-erb-B-2, or c-myc mutations, to survive by evading apoptosis [15]. Furthermore, these expression patterns may explain the controversial Bcl-2 data generated by previous studies. Most studies investigating Bcl-2 expression in ChCs have reported varying degrees of Bcl-2 protein expression [8, 16, 18, 23, 24]. A study by Charlotte et al. [8] showed that the Bcl-2 protein is expressed by small bile duct epithelia in normal livers but not by hepatocytes or cells of the large duct. These authors suggested that the Bcl-2 protein is a diagnostic marker to distinguish ChC from hepatocellular carcinoma. A study by Skopelitou et al. [16] found Bcl-2 expression in normal small bile duct epithelia and in all cases of ChC, whereas it was not detected in neoplastic and dysplastic bile ducts, or normal hepatocytes. A study by Skopelitou et al. [16] also suggested that Bcl-2 appears to be a marker that distinguishes hepatocellular carcinoma from ChC. However, Bcl-2 expression in ChCs is also very controversial. A study by Guo et al. [23] reported that 21 of 29 ChCs express the Bcl-2 protein (72.4%). In contrast, a study by Ito et al. [18] reported that only 31.7% (13/41) of ChCs are positive for Bcl-2 and that Bcl-2 expression was found in only 23.6% of ChCs and exhibited a strong association with tumor localization. Furthermore, some studies have reported that none of the ChC samples express Bcl-2 protein [17, 19, 20]. A study by Arora et al. [17] reported that most ChC cases did not express immunohistochemically detectable amounts of Bcl-2 protein and that its function could be performed by other proteins such as Bcl-X_L_. A study by Terada and Nakanuma [20] also showed low or negative expression of Bcl-2 in bile duct epithelia and ChCs. In our paper, only 14.1% (690/4875) of small bile duct epithelia at the carcinogenic phase were positive for Bcl-2, indicating that the role of Bcl-2 in ChCs is not very significant.

 We were interested in determining what other molecules could play an important role in cancer cell survival and that are essential for tumor growth. Thus, we focused on the expression pattern of thioredoxin (Trx), a redox component and another apoptosis-inhibiting factor, during various stages of hamster and human cholangiocarcinogenesis [21]. Trx was heterogeneously expressed in hyperplastic bile ducts with morphological alterations, which was similar to the Bcl-2 expression pattern. However, unlike Bcl-2, high Trx expression continued consistently into the carcinogenic phase; Trx was overexpressed in hamster and human ChCs. We were unable to determine if an interacting relationship occurred between Bcl-2 and Trx during cholangiocarcinogenesis, but compensatory relationships between these two proteins were recently reported by Li et al. [25]. According to that study, Trx is correspondingly upregulated with Bcl-2 downregulation, following transfection of neuroblastoma cells with antisense Bcl-2. Further studies are needed to verify whether a similar relationship between Trx and Bcl-2 is involved in cholangiocarcinogenesis.

 Why such a discrepancy in Bcl-2 expression is present in human ChCs as well as normal bile ducts in previous studies is still unclear but may have arisen because of the different methods used or differences in the ChC specimens. In our study, the same procedures were applied to all specimens, including fixation, tissue processing, and immunohistochemistry. The specificity of the Bcl-2 antibody used in the hamster livers was also validated by western blotting.

 In summary, Bcl-2 was heterogeneously overexpressed in a limited number of dysplastic bile duct epithelial cells in a hamster ChC model, suggesting that the protein plays some role in cell survival of transformed bile duct cells. However, its role in the carcinogenic phase of ChCs was not significant in the present hamster ChC model.
